# Crystal structure and hydrogen bonding in *N*-(1-de­oxy-β-d-fructo­pyranos-1-yl)-2-amino­isobutyric acid

**DOI:** 10.1107/S2056989017018060

**Published:** 2018-01-01

**Authors:** Valeri V. Mossine, Charles L. Barnes, Thomas P. Mawhinney

**Affiliations:** a Department of Biochemistry, University of Missouri, Columbia, MO 65211, USA; b Department of Chemistry, University of Missouri, Columbia, MO 65211, USA

**Keywords:** crystal structure, fructosamine, Maillard reaction, d-fructose-2-amino­isobutyric acid, hydrogen bonding, Hirshfeld surface analysis

## Abstract

The title compound, alternatively called d-fructose-2-amino­isobutyric acid (FruAib), C_10_H_19_NO_7_, (I), crystallizes exclusively in the β-pyran­ose form, with two conformationally non-equivalent mol­ecules [(I*A*) and (I*B*)] in the asymmetric unit. In solution, FruAib establishes an equilibrium, with 75.6% of the population consisting of β-pyran­ose, 10.4% β-furan­ose, 10.1% α-furan­ose, 3.0% α-pyran­ose and <0.7% the acyclic forms. The carbohydrate ring in (I) has the normal ^2^ *C*_5_ chair conformation and the amino acid portion is in the zwitterion form. Bond lengths and valence angles compare well with the average values from related pyran­ose structures. All carboxyl, hy­droxy and ammonium groups are involved in hydrogen bonding and form a three-dimensional network of infinite chains that are connected through homodromic rings and short chains. Intra­molecular hydrogen bonds bridge the amino acid and sugar portions in both mol­ecules. A comparative Hirshfeld surfaces analysis of FruAib and four other sugar–amino acids suggests an increasing role of intra­molecular heteroatom inter­actions in crystal structures with an increasing proportion of C—H bonds.

## Abstract 2

The asymmetric unit contains two conformationally unequal zwitterion mol­ecules that differ in the intra­molecular hydrogen-bonding patterns. The ^2^C_5_ β-fructo­pyran­ose conformation also dominates in the compound’s solution.

## Chemical context   

**Figure 1:**
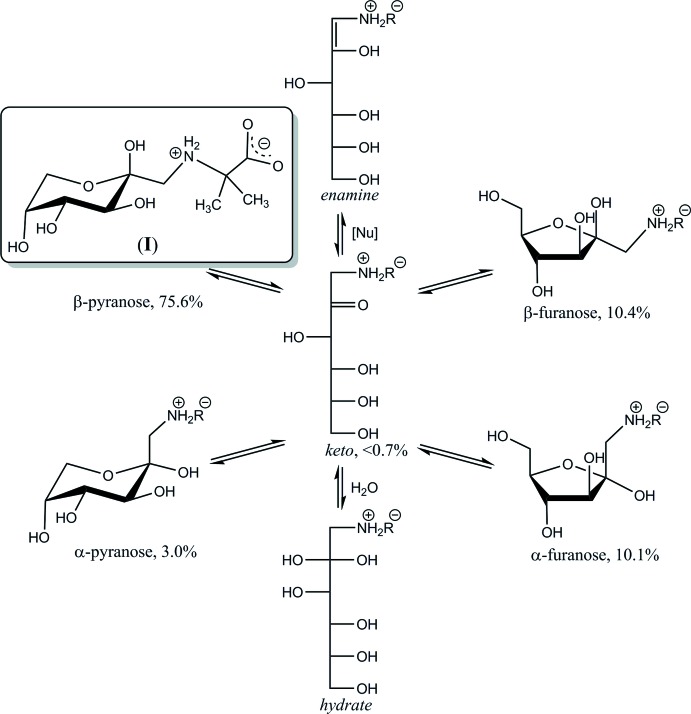
Equilibrium in aqueous solution of (I), at 293 K and pH 6.

d-Fructose-amino acids are derivatives of fructosamine and represent the major fraction of the early Maillard reaction products which form non-enzymatically both in processed foods and *in vivo* (Mossine & Mawhinney, 2010 ▸). Naturally occurring d-fructose-amino acids act as inter­mediates in the formation of food aroma and colour, while elevated fructosamine content in humans has been linked to the development of diabetic complications and tissue damage. Synthetic fructo­samine derivatives have been offered as lectin blockers and anti­oxidants that might stimulate immune system (Tarnawski, Kuliś-Orzechowska & Szelepin, 2007 ▸), be potentially useful in prevention of cancer metastasis (Mossine *et al.*, 2010 ▸), or neuroinflammation (Song *et al.*, 2016 ▸). The chemical and biological reactivity of fructosamines stems from their structural instability. Thus, in solutions, fructosamine derivatives rapidly establish a equilibrium between several cyclic and acyclic forms (Kaufmann *et al.*, 2016 ▸), as exemplified in Fig. 1 ▸ for the title compound. The acyclic tautomers, while present in minute (<1%) proportions, are responsible for chemical transformations of fructosamines in numerous redox, isomerization, or degradation reactions. The cyclic conformers are responsible for the carbohydrate recognition by proteins such as lectins, transporters or enzymes, and thus define a number of biological activities of fructosamines (Mossine & Mawhinney, 2010 ▸).
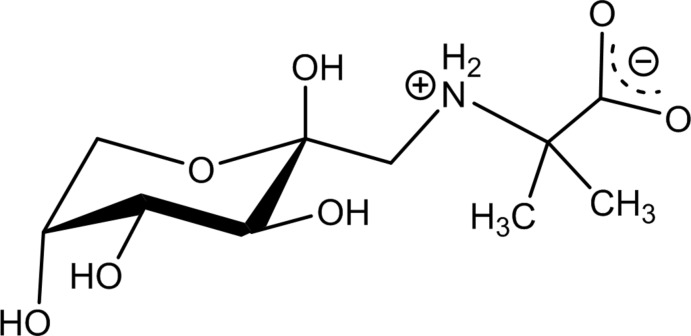


As a part of our structure–activity studies, we have prepared d-fructose-2-amino­isobutyric acid (FruAib), a structural analogue of an efficient blocker of galectins-1, −3 and −4, d-fructose-l-leucine (Mossine *et al.*, 2008 ▸). In this work, we report on the mol­ecular and crystal structure of FruAib, C_10_H_19_NO_7_ (I), with an emphasis on hydrogen-bonding patterns in the structure. A comparative Hirshfeld surfaces analysis of FruAib and four other sugar-amino acids is also completed.

## Structural commentary   

**Figure 2:**
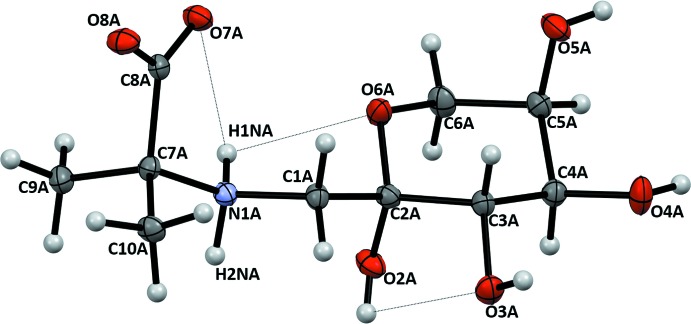
Atomic numbering and displacement ellipsoids at the 50% probability level for mol­ecule (I*A*). Intra­molecular N—H⋯O and O—H⋯O inter­actions are shown as dotted lines.

Crystalline FruAib has two conformationally nonequivalent mol­ecules, (I*A*) and (I*B*), in the asymmetric unit. The mol­ecular structures and atomic numbering are shown in Figs. 2 ▸ and 3 ▸. The mol­ecules may be considered as conjugates of a carbohydrate, 1-amino-1-de­oxy-d-fructose, and an amino acid, 2-amino­isobutyric acid, which are joined through the common amino group. The β-d-fructo­pyran­ose rings of the carbohydrate portions in both (I*A*) and (I*B*) exist in the ^2^ *C*_5_ chair conformation, with puckering parameters *Q* = 0.582 Å, *q* = 177.7°, and *f* = 224° for (I*A*) and *Q* =0.565 Å, *q* = 175.5°, and *f* = 268° for (I*B*). These parameters correspond to a conformation with the lowest energy possible for fructose (French *et al.*, 1997 ▸), with (I*B*) providing a better fit. The bond distances and the valence angles are close to the average values for a number of crystalline pyran­ose structures (Jeffrey & Taylor, 1980 ▸). In the solution of FruAib, the β-d-pyran­ose anomer dominates the equilibrium, at 76.6%, as follows from the ^13^C NMR spectrum (Fig. 1 ▸, Supporting Table S1). In the ^1^H NMR spectrum of the major anomer (see Section 5), the vicinal proton–proton coupling constants *J*_3,4_ = 9.8 Hz and *J*_4,5_ = 3.4 Hz indicate H4 is in the *trans* disposition to H3 and in the *gauche* disposition to H5. Hence, the predominant conformation of FruAib in solution is the ^2^ *C*_5_ β-d-fructo­pyran­ose, as well.

The amino acid portions of both (I*A*) and (I*B*) are in the zwitterion form with a positively charged tetra­hedral secondary ammonium nitro­gen and a negatively charged deprotonated carboxyl group. Each mol­ecule has three intra­molecular inter­actions (Table 1 ▸), two of which bridge the carboxyl­ate, ammonium, and the carbohydrate portions of the mol­ecules. The intra­molecular hydrogen-bonding patterns differ in the mol­ecules. Thus, in (I*B*), the string of short heteroatom contacts stretches from O4*B* through O7*B* and can be denoted in terms of the 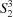
(5) pattern descriptor. In (I*A*), the intra­molecular hydrogen bonding is fragmented between the shorter zwitterionic bridge O7*A*⋯H1N*A*⋯O6*A* [the 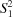
(3) pattern] and the O2*A*—H⋯O3*A* contact. In the ^1^H NMR spectrum of FruAib (see Section 5), the two protons attached to C1 produce two distinct signals at 3.297 and 3.210 ppm, with *J*_1A,1B_ = −12.7 Hz. The inequality of these protons indicates restricted rotation around the C1—C2 and C1—C7 bonds, thus suggesting that the intra­molecular hydrogen bonds retain the structure in solution (Mossine *et al.*, 1994 ▸). There are non-equivalences in carboxyl­ate C—O distances that are observed in both mol­ecules and which could be attributed to unequal participation of the oxygen atoms in hydrogen bonding. In (I*A*), O8*A* is involved in a three-center hydrogen-bonding inter­action, with H⋯O8*A* distances of 1.79 and 1.98 Å, while for the O7*A* inter­action, the distances are 1.91 and 2.30 Å (Table 1 ▸), which explains the elongation of the C8*A*—O8*A* bond (1.260 Å), as compared to the C8*A*—O7*A* distance (1.249 Å). Similar considerations can be applied to (I*B*), where O7*B* is involved in two short heteroatom contacts and O8*B* participates in only one (Table 1 ▸), hence the difference in the C8*B*—O7*B* (1.263 Å) and C8*B*—O8*B* (1.241 Å) bond lengths.

## Supra­molecular features   

**Figure 4:**
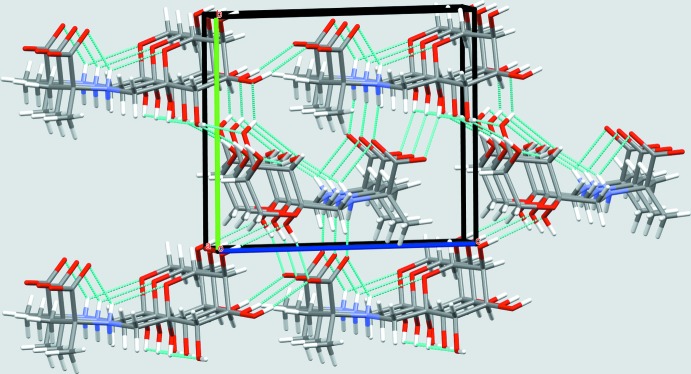
The mol­ecular packing in (I). Color code for crystallographic axes: red −*a*, green −*b*, blue −*c*. Hydrogen bonds are shown as cyan dotted lines.

**Figure 5:**
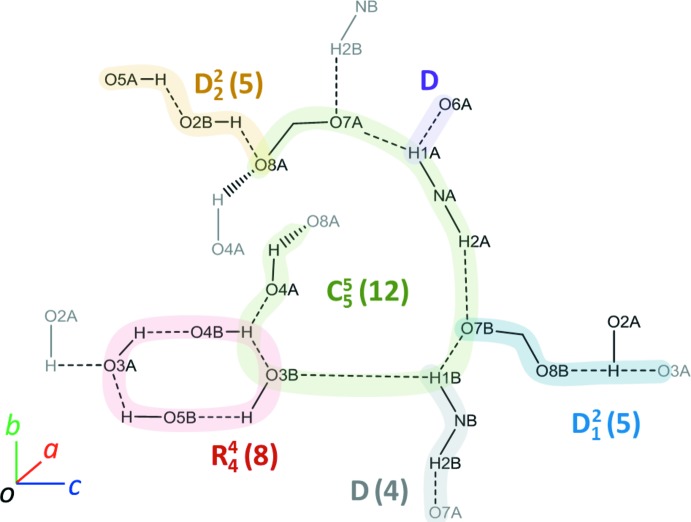
Hydrogen-bond patterns in the crystal structure of (I).

FruAib crystallizes in the triclinic space group *P*1, with two non-equivalent mol­ecules per unit cell. The mol­ecular packing of (I) features infinite chains of hydrogen bonds spiralling along the *a* axis (Fig. 4 ▸). The basic hydrogen-bonding patterns are depicted in Fig. 5 ▸ and include the main infinite chain pattern 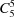
(12); in the crystal, these infinite chains are connected through homodromic rings [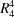
(8)] and short chains [*D*_1_^2^(5) and *D*(4)]. Thus, hydrogen bonds form a three-dimensional network of short heteroatomic contacts throughout the crystal of (I). In addition, there are a number of close C—H⋯O contacts that may qualify as weak hydrogen bonds (Table 2 ▸). Inter­estingly, mol­ecule (I*A*) provides most of donors for these contacts.

## Database survey   

Search of SciFinder, Google Scholar, and the Cambridge Structural Database (Groom *et al.*, 2016 ▸) by both structure and chemical names revealed no previous structural description of d-fructose-2-amino­isobutyric acid: thus the compound appears to be novel. The d-fructosamine portion of the mol­ecule is more inter­esting for a structure comparison survey due to its conformational instability and practical significance to food and health sciences. The most closely related structures are d-fructose-glycine (FruGly, CCDC 1307697; Mossine *et al.*, 1995 ▸) and d-fructose-l-proline (FruPro, CCDC 628806, 628807, 631528; Tarnawski, Ślepokura *et al.*, 2007 ▸). These d-fructose-amino acids crystallize in the ^2^ *C*_5_ β-pyran­ose conformations and exist as zwitterions as well, with the intra­molecular hydrogen bonding that necessarily involves the amino acid carboxyl­ate, the ammonium group and one hy­droxy group donated by the carbohydrate moiety. However, none of these structures features the involvement of the pyran­ose ring O6 in the intra­molecular hydrogen bonding found in (I*A*). On the other hand, (I*B*) is structurally close to both FruGly (Mossine *et al.*, 1995 ▸) and FruPro (Tarnawski, Ślepokura *et al.*, 2007 ▸). In the mol­ecules, the conformations around the C1—C2 bond are *trans–gauche*, with respective values of the N—C1—C2—O6 torsion angle falling into the 165–177° range and are stabilized with the similar intra­molecular hydrogen-bonding pattern O3⋯H1*N*⋯O7.

**Figure 6:**
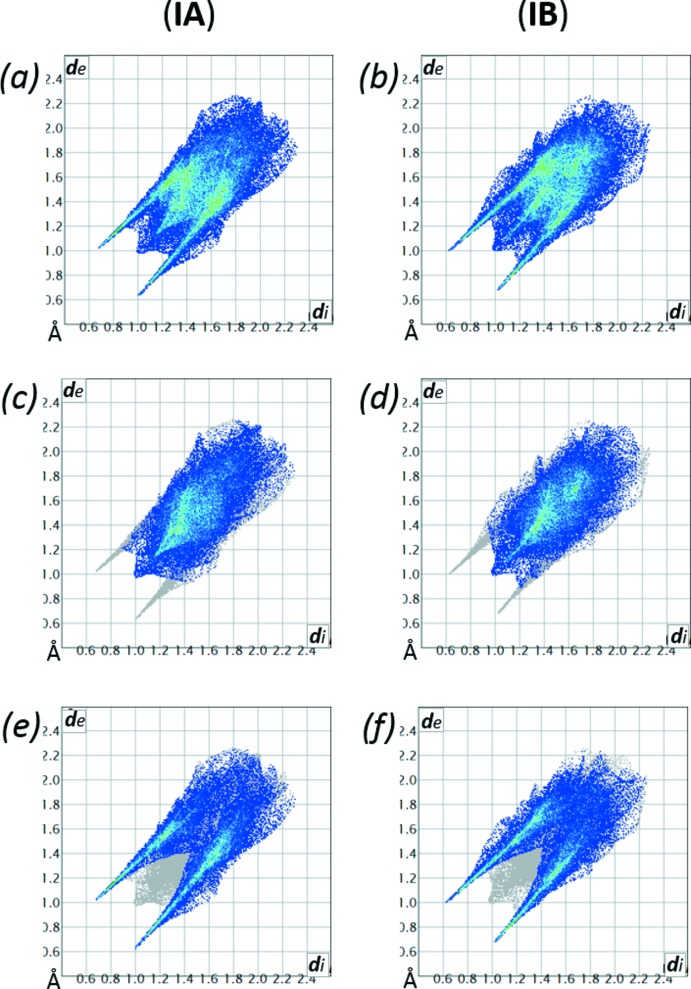
Two-dimensional fingerprint plots produced for the Hirshfeld surfaces of (I*A*) and (I*B*). The full plots for (I*A*) and (I*B*) are shown in (*a*) and (*b*), respectively. Contributions to the plots from the H⋯H contacts are shown in (*c*) and (*d*) and the contributions from the O⋯H/H⋯O contacts are depicted in (*e*) and (*f*).

A compendium of structures close to (I) is presented in Table 3 ▸. In addition to FruPro and FruGly, two structures isomeric to FruGly were included: d-galactose-glycine (GalGly, CCDC123625; Mossine *et al.*, 1996 ▸) and d-glucose-glycine (GlcGly, CCDC123624; Mossine *et al.*, 1996 ▸). In sugar-amino acids, as demonstrated in Table 3 ▸, an increase in the proportion of C—H bonds leads to an increase in number of intra­molecular hydrogen bonds. Such tendency towards the ‘inter­nalization’ of hydrogen bonding was also noticed as a result of a comparative analysis of the ‘fingerprint plots’ based on the calculated Hirshfeld surfaces (Spackman & Jayatilaka, 2009 ▸) and delineated for the O⋯H/H⋯O contacts (Fig. 6 ▸). Table 3 ▸ lists the relative abundances of these contacts calculated for (I*A*), (I*B*) and structurally close sugar-amino acids. There is an obvious trend towards decrease in the proportion of inter­molecular O⋯H contacts as the number of the C—H bonds in the structure increases, although a total number of hydrogen-bonds per mol­ecule increases as well.

## Synthesis and crystallization   

2-Amino­isobutyric acid (2.1 g, 0.02 mol), d-glucose (9 g, 0.05 mol), and sodium acetate (0.82 g, 0.01 mol) were dissolved in 100 ml of a methanol/glycerol (3:1) mixture and refluxed for 3 h. The reaction progress was monitored by TLC on silica. The reaction mixture was diluted with 900 ml of water and passed through a column charged with 80 ml of Amberlite IRN-77 (H^+^-form). The target compound was then eluted with 0.2 *M* pyridine, and fractions containing pure FruAib were pooled and evaporated. The residue was redissolved in 100 ml of water, decolorized with 0.5 g of charcoal and evaporated to a syrup. The latter was dissolved in 30 ml of ethanol and made nearly cloudy with dropwise addition of acetone. Crystallization occurred within a week at room temperature. Yield 2.0 g (38%, based on starting Aib). Major (β-pyran­ose anomer) peaks (ppm) in the ^13^C NMR spectrum in D_2_O: 179.35 (C8); 98.33 (C2); 72.39 (C4); 72.21 (C3); 71.79 (C5); 67.00 (C7); 66.68 (C6); 51.72 (C1); 24.66, 24.47 (C9, C10). See Supporting Table S1 for minor peak assignments in the spectrum. Major signals (ppm) and resolved coupling constants (Hz) in the ^1^H NMR spectrum: 4.038 (*dd*, H6*B*); 4.021 (*m*, H5); 3.903 (*dd*, H4); 3.784 (*d*, H3); 3.775 (*dd*, H6*A*); 3.297 (*d*, H1*B*); 3.210 (*d*, H1*A*); 1.517 (*s*, 3H10); 1.512 (*s*, 3H9); *J*_1A,1B_ = −12.7; *J*_3,4_ = 9.8; *J*_4,5_ = 3.4; *J*_5,6A_ = 1.3; *J*_6A,6B_ = −12.9.

## Refinement   

Crystal data, data collection and structure refinement details are summarized in Table 4 ▸. Hy­droxy and nitro­gen-bound H atoms were located in difference-Fourier analyses and were allowed to refine fully. Other H atoms were placed at calculated positions and treated as riding, with C—H = 0.98 Å (meth­yl), 0.99 Å (methyl­ene) or 1.00 Å (methine) and with *U*_iso_(H) = 1.2*U*_eq_(methine or methyl­ene) or 1.5*U*_eq_(meth­yl). As a result of the unrealistic value obtained for the Flack absolute structure parameter [−0.5 (3) for 2254 quotients (Parsons *et al.*, 2013 ▸)], the absolute configuration of the ring system (2*R*,3*S*,4*R*,5*R*) was assigned on the basis of the known configuration for the starting compound d-glucose (McNaught, 1996 ▸).

## Supplementary Material

Crystal structure: contains datablock(s) I. DOI: 10.1107/S2056989017018060/zs2396sup1.cif

Structure factors: contains datablock(s) I. DOI: 10.1107/S2056989017018060/zs2396Isup2.hkl

CCDC reference: 1583254

Additional supporting information:  crystallographic information; 3D view; checkCIF report

## Figures and Tables

**Figure 3:**
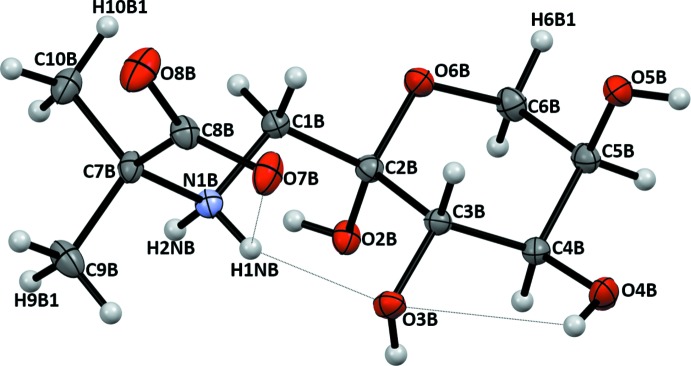
Atomic numbering and displacement ellipsoids at the 50% probability level for mol­ecule (I*B*). Intra­molecular N—H⋯O and O—H⋯O inter­actions are shown as dotted lines.

**Table 1:** Hydrogen-bond geometry (Å, °)

*D*—H⋯*A*	*D*—H	H⋯*A*	*D*⋯*A*	*D*—H⋯*A*
N1*A*—H1*NA*⋯O6*A*	0.86 (3)	2.40 (3)	2.813 (3)	110 (2)
N1*A*—H1*NA*⋯O7*A*	0.86 (3)	2.30 (3)	2.674 (2)	107 (2)
O2*B*—H2*OB*⋯O8*A* ^i^	0.84 (3)	1.78 (3)	2.596 (3)	165 (3)
N1*A*—H2*NA*⋯O7*B*	0.98 (3)	1.78 (3)	2.743 (3)	169 (3)
O5*A*—H5*OA*⋯O2*B* ^ii^	0.76 (4)	2.14 (4)	2.886 (3)	168 (4)
O5*B*—H5*OB*⋯O3*A* ^iii^	0.83 (4)	1.99 (4)	2.804 (3)	165 (3)
O2*A*—H2*OA*⋯O3*A*	0.82 (4)	2.62 (3)	2.847 (2)	97 (3)
O3*A*—H3*OA*⋯O4*B* ^iv^	0.78 (4)	2.08 (4)	2.785 (3)	149 (3)
O4*A*—H4*OA*⋯O8*A* ^v^	0.84 (4)	2.00 (4)	2.822 (3)	170 (4)
O2*A*—H2*OA*⋯O8*B*	0.82 (4)	1.87 (4)	2.657 (3)	161 (4)
O4*B*—H4*OB*⋯O3*B*	0.84 (4)	2.51 (4)	2.886 (2)	108 (3)
O4*B*—H4*OB*⋯O4*A* ^vi^	0.84 (5)	2.14 (5)	2.864 (3)	145 (5)
N1*B*—H2*NB*⋯O7*A* ^i^	0.90 (3)	1.91 (3)	2.795 (3)	168 (3)
N1*B*—H1*NB*⋯O3*B*	0.90 (4)	2.02 (4)	2.800 (3)	144 (3)
N1*B*—H1*NB*⋯O7*B*	0.90 (4)	2.40 (3)	2.681 (3)	100 (2)
O3*B*—H3*OB*⋯O5*B* ^vii^	0.86 (4)	1.92 (4)	2.717 (3)	154 (4)

Symmetry codes: (i) 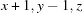; (ii) 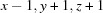; (iii) 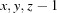; (iv) 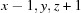; (v) 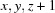; (vi) 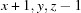; (vii) 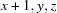.

**Table 2:** Suspected C—H⋯O contacts (Å, °) in (I)

*D*—H⋯*A*	*D*—H	H⋯*A*	*D*⋯*A*	*D*—H⋯*A*	Symmetry code
C1*A*—H1*A*1⋯O3*A*	0.99	2.56	2.909 (3)	101	
C4*A*—H4*A*⋯O4*B*	1.00	2.63	3.608 (3)	167	*x*, *y*, *z* + 1
C9*A*—H9*A*1⋯O8*A*	0.98	2.55	3.313 (3)	135	*x* + 1, *y*, *z*
C9*A*—H9*A*3⋯O3*B*	0.98	2.66	3.575 (3)	156	
C9*A*—H9*A*3⋯O7*B*	0.98	2.68	3.381 (3)	129	
C10*A*—H10*A*⋯O7*B*	0.98	2.72	3.451 (3)	132	
C10*A*—H10*B*⋯O3*B*	0.98	2.64	3.076 (3)	107	*x* − 1, *y*, *z*
C5*B*—H5*B*⋯O8*A*	1.00	2.41	3.355 (3)	156	*x*, *y*, *z* − 1
C6*B*—H6*B*2⋯O5*A*	0.99	2.61	3.556 (3)	161	*x* + 1, *y* − 1, *z*
C10*B*—H10*E*⋯O5*A*	0.98	2.71	3.517 (3)	140	*x* + 1, *y* − 1, *z*
C10*B*—H10*F*⋯O7*A*	0.98	2.70	3.443 (3)	133	*x* + 1, *y* − 1, *z*

**Table 3:** Hydrogen bonding and contributions of the O⋯H/H⋯O contacts to the Hirshfeld surfaces of sugar-amino acids Notes: (*) All sugar-amino acids are in the pyran­ose form and all have four hy­droxy, one carboxyl and one ammonium group, and one pyran­ose ring oxygen; (**) hydrogen-bond selection criteria: *D*⋯*A* < 2.9 Å; H⋯*A* < 2.7 Å; *D*—H⋯*A* >95°.

Structure*	No. of CH/CH_2_/CH_3_ groups (total C—H)	No. of intra/inter hydrogen-bonds**	% of O⋯H/H⋯O contacts on Hirshfeld surface	Reference
GalGly	3/3/0 (9)	2/6	55.7	Mossine *et al.* (1996 ▸)
GlcGly	3/3/0 (9)	3/6	57.6	Mossine *et al.* (1996 ▸)
FruGly	3/3/0 (9)	2/6	51.6	Mossine *et al.* (1995 ▸)
FruAib (IA)	3/2/2 (13)	3/5	44.0	This work
FruAib (IB)	3/2/2 (13)	3/5	45.9	This work
FruPro·H_2_O	4/5/0 (14)	3/6	49.2	Mossine *et al.* (2007 ▸)
FruPro·2H_2_O	4/5/0 (14)	3/6	49.3	Tarnawski, Ślepokura *et al.* (2007 ▸)
FruPro·MeOH	4/5/1 (17)	4/5	40.2	Tarnawski, Ślepokura *et al.* (2007 ▸)

**Table 4:** Experimental details

Crystal data	
Chemical formula	C_10_H_19_NO_7_
*M*_r_	265.26
Crystal system, space group	Triclinic, *P*1
Temperature (K)	100
*a*, *b*, *c* (Å)	5.8008 (19), 9.636 (3), 10.676 (4)
α, β, γ (°)	87.766 (3), 86.330 (4), 82.042 (4)
*V* (Å^3^)	589.5 (3)
*Z*	2
Radiation type	Mo *K*α
μ (mm^−1^)	0.13
Crystal size (mm)	0.25 × 0.20 × 0.08

Data collection	
Diffractometer	Bruker APEXII CCD area detector
Absorption correction	Multi-scan (*SADABS*; Sheldrick, 2003 ▸)
*T*_min_, *T*_max_	0.86, 0.99
No. of measured, independent and observed [*I* > 2σ(*I*)] reflections	6952, 5160, 4927
*R*_int_	0.022
(sin θ/λ)_max_ (Å^−1^)	0.652

Refinement	
*R*[*F* ^2^ > 2σ(*F* ^2^)], *wR*(*F* ^2^), *S*	0.033, 0.081, 1.03
No. of reflections	5160
No. of parameters	377
No. of restraints	3
H-atom treatment	H atoms treated by a mixture of independent and constrained refinement
Δρ_max_, Δρ_min_ (e Å^−3^)	0.30, −0.22
Absolute structure	Flack *x* determined using 2254 quotients [(*I* ^+^)−(*I* ^−^)]/[(*I* ^+^)+(*I* ^−^)] (Parsons *et al.*, 2013 ▸)
Absolute structure parameter	−0.5 (3)

Computer programs: *APEX2* and *SAINT* (Bruker, 1998 ▸), *SHELXS97* (Sheldrick, 2008 ▸), *SHELXL2017* (Sheldrick, 2015 ▸), *X-SEED* (Barbour, 2001 ▸), *Mercury* (Macrae *et al.*, 2008 ▸), *CIFTAB* (Sheldrick, 2008 ▸) and *publCIF* (Westrip, 2010 ▸).

## supplementary crystallographic information

### Crystal data

**Table:**

C_10_H_19_NO_7_	*Z* = 2
*M**_r_* = 265.26	*F*(000) = 284
Triclinic, *P*1	*D*_x_ = 1.494 Mg m^−^^3^
*a* = 5.8008 (19) Å	Mo *K*α radiation, λ = 0.71073 Å
*b* = 9.636 (3) Å	Cell parameters from 4131 reflections
*c* = 10.676 (4) Å	θ = 2.8–27.6°
α = 87.766 (3)°	µ = 0.13 mm^−^^1^
β = 86.330 (4)°	*T* = 100 K
γ = 82.042 (4)°	Plate, colourless
*V* = 589.5 (3) Å^3^	0.25 × 0.20 × 0.08 mm

### Data collection

**Table:**

Bruker APEXII CCD area detector diffractometer	4927 reflections with *I* > 2σ(*I*)
ω scans	*R*_int_ = 0.022
Absorption correction: multi-scan (SADABS; Sheldrick, 2003)	θ_max_ = 27.6°, θ_min_ = 1.9°
*T*_min_ = 0.86, *T*_max_ = 0.99	*h* = −7→7
6952 measured reflections	*k* = −12→12
5160 independent reflections	*l* = −13→13

### Refinement

**Table:**

Refinement on *F*^2^	Hydrogen site location: mixed
Least-squares matrix: full	H atoms treated by a mixture of independent and constrained refinement
*R*[*F*^2^ > 2σ(*F*^2^)] = 0.033	*w* = 1/[σ^2^(*F*_o_^2^) + (0.0439*P*)^2^ + 0.1*P*] where *P* = (*F*_o_^2^ + 2*F*_c_^2^)/3
*wR*(*F*^2^) = 0.081	(Δ/σ)_max_ < 0.001
*S* = 1.03	Δρ_max_ = 0.30 e Å^−^^3^
5160 reflections	Δρ_min_ = −0.22 e Å^−^^3^
377 parameters	Absolute structure: Flack *x* determined using 2254 quotients [(*I*^+^)-(*I*^-^)]/[(*I*^+^)+(*I*^-^)] (Parsons *et al.*, 2013)
3 restraints	Absolute structure parameter: −0.5 (3)

### Special details

**Table:**

Geometry. All esds (except the esd in the dihedral angle between two l.s. planes) are estimated using the full covariance matrix. The cell esds are taken into account individually in the estimation of esds in distances, angles and torsion angles; correlations between esds in cell parameters are only used when they are defined by crystal symmetry. An approximate (isotropic) treatment of cell esds is used for estimating esds involving l.s. planes.

### Fractional atomic coordinates and isotropic or equivalent isotropic displacement parameters (Å^2^)

**Table:**

	*x*	*y*	*z*	*U*_iso_*/*U*_eq_	
N1A	0.4852 (3)	0.7001 (2)	0.59886 (18)	0.0140 (4)	
C1A	0.2937 (4)	0.6814 (2)	0.6955 (2)	0.0155 (4)	
H1A1	0.258387	0.583790	0.696105	0.019*	
H1A2	0.150865	0.745067	0.675402	0.019*	
O2A	0.5882 (3)	0.6392 (2)	0.84699 (16)	0.0201 (4)	
C2A	0.3701 (4)	0.7143 (2)	0.8240 (2)	0.0149 (4)	
O3A	0.1611 (3)	0.54217 (18)	0.93040 (17)	0.0200 (4)	
C3A	0.1796 (4)	0.6889 (2)	0.9256 (2)	0.0160 (4)	
H3A	0.027565	0.743040	0.902764	0.019*	
O4A	0.0554 (3)	0.7173 (2)	1.14090 (17)	0.0235 (4)	
C4A	0.2446 (4)	0.7366 (2)	1.0515 (2)	0.0166 (4)	
H4A	0.388769	0.676038	1.077616	0.020*	
O5A	0.0858 (3)	0.9847 (2)	1.01890 (19)	0.0256 (4)	
C5A	0.2914 (4)	0.8897 (2)	1.0402 (2)	0.0173 (5)	
H5A	0.356155	0.914273	1.119767	0.021*	
O6A	0.3959 (3)	0.85831 (17)	0.81688 (15)	0.0185 (3)	
C6A	0.4700 (4)	0.9059 (3)	0.9321 (2)	0.0208 (5)	
H6A1	0.492827	1.005718	0.921278	0.025*	
H6A2	0.621286	0.851162	0.951912	0.025*	
O7A	0.2928 (3)	0.95121 (17)	0.51308 (16)	0.0205 (4)	
C7A	0.4156 (4)	0.7112 (2)	0.4646 (2)	0.0140 (4)	
O8A	0.1246 (3)	0.86077 (17)	0.35794 (16)	0.0204 (4)	
C8A	0.2629 (4)	0.8535 (2)	0.4449 (2)	0.0147 (4)	
C9A	0.6394 (4)	0.7138 (3)	0.3809 (2)	0.0190 (5)	
H9A1	0.718756	0.791634	0.404061	0.028*	
H9A2	0.600415	0.726203	0.292846	0.028*	
H9A3	0.742340	0.625157	0.392235	0.028*	
C10A	0.2936 (4)	0.5854 (2)	0.4386 (2)	0.0176 (5)	
H10A	0.386894	0.499035	0.467924	0.026*	
H10B	0.276226	0.581322	0.348147	0.026*	
H10C	0.139289	0.595194	0.483013	0.026*	
N1B	0.9798 (3)	0.1994 (2)	0.51896 (19)	0.0148 (4)	
C1B	0.7661 (4)	0.1679 (2)	0.4597 (2)	0.0162 (4)	
H1B1	0.628379	0.230040	0.494220	0.019*	
H1B2	0.742314	0.069761	0.481061	0.019*	
O2B	0.9828 (3)	0.10708 (18)	0.26199 (16)	0.0177 (3)	
C2B	0.7867 (4)	0.1888 (2)	0.3164 (2)	0.0153 (4)	
O3B	1.0020 (3)	0.39052 (17)	0.31557 (16)	0.0179 (3)	
C3B	0.7993 (4)	0.3425 (2)	0.2743 (2)	0.0147 (4)	
H3B	0.660381	0.401144	0.314343	0.018*	
O4B	0.7785 (3)	0.50320 (19)	0.09278 (18)	0.0220 (4)	
C4B	0.7840 (4)	0.3599 (2)	0.1331 (2)	0.0157 (4)	
H4B	0.923045	0.303446	0.090998	0.019*	
O5B	0.3592 (3)	0.39757 (19)	0.13899 (18)	0.0212 (4)	
C5B	0.5615 (4)	0.3074 (3)	0.0932 (2)	0.0178 (5)	
H5B	0.564638	0.306047	−0.000506	0.021*	
O6B	0.5737 (3)	0.15023 (18)	0.28044 (16)	0.0184 (3)	
C6B	0.5487 (4)	0.1611 (3)	0.1472 (2)	0.0196 (5)	
H6B1	0.396939	0.132758	0.129061	0.023*	
H6B2	0.673214	0.095284	0.105088	0.023*	
O7B	0.7832 (3)	0.45290 (18)	0.59469 (17)	0.0248 (4)	
C7B	0.9506 (4)	0.2216 (2)	0.6593 (2)	0.0158 (4)	
O8B	0.6794 (4)	0.3715 (2)	0.78640 (18)	0.0301 (5)	
C8B	0.7867 (4)	0.3608 (2)	0.6822 (2)	0.0174 (5)	
C9B	1.1926 (4)	0.2407 (3)	0.7005 (3)	0.0241 (5)	
H9B1	1.299035	0.153209	0.689055	0.036*	
H9B2	1.180988	0.264886	0.789159	0.036*	
H9B3	1.252503	0.316092	0.649424	0.036*	
C10B	0.8641 (5)	0.0944 (3)	0.7269 (2)	0.0221 (5)	
H10D	0.705980	0.087100	0.702985	0.033*	
H10E	0.862555	0.104968	0.817864	0.033*	
H10F	0.968250	0.009301	0.703146	0.033*	
H1NA	0.536 (5)	0.778 (3)	0.613 (3)	0.016 (7)*	
H2OB	1.012 (6)	0.032 (3)	0.303 (3)	0.021 (7)*	
H2NA	0.599 (5)	0.616 (3)	0.607 (3)	0.019 (7)*	
H5OA	0.040 (6)	1.015 (4)	1.082 (4)	0.027 (9)*	
H5OB	0.311 (6)	0.453 (4)	0.082 (4)	0.032 (9)*	
H3OA	0.030 (7)	0.539 (4)	0.951 (3)	0.029 (9)*	
H4OA	0.086 (7)	0.750 (4)	1.208 (4)	0.045 (11)*	
H2OA	0.590 (6)	0.555 (4)	0.840 (3)	0.033 (9)*	
H4OB	0.852 (10)	0.549 (5)	0.138 (5)	0.074 (15)*	
H2NB	1.090 (5)	0.124 (3)	0.507 (3)	0.017 (7)*	
H1NB	1.021 (6)	0.276 (4)	0.478 (3)	0.029 (8)*	
H3OB	1.114 (7)	0.365 (4)	0.261 (4)	0.045 (10)*	

### Atomic displacement parameters (Å^2^)

**Table:**

	*U*^11^	*U*^22^	*U*^33^	*U*^12^	*U*^13^	*U*^23^
N1A	0.0143 (9)	0.0143 (9)	0.0131 (9)	−0.0003 (7)	−0.0011 (7)	−0.0013 (7)
C1A	0.0159 (10)	0.0182 (11)	0.0124 (10)	−0.0020 (8)	0.0001 (8)	−0.0009 (8)
O2A	0.0173 (8)	0.0212 (9)	0.0204 (9)	0.0038 (7)	−0.0035 (6)	−0.0043 (7)
C2A	0.0162 (10)	0.0133 (10)	0.0150 (10)	−0.0010 (8)	−0.0018 (8)	−0.0020 (8)
O3A	0.0232 (9)	0.0181 (8)	0.0197 (8)	−0.0070 (7)	0.0012 (7)	−0.0010 (7)
C3A	0.0196 (11)	0.0155 (10)	0.0129 (10)	−0.0019 (8)	0.0001 (8)	−0.0012 (8)
O4A	0.0285 (9)	0.0306 (10)	0.0135 (8)	−0.0125 (8)	0.0040 (7)	−0.0050 (7)
C4A	0.0183 (11)	0.0174 (11)	0.0144 (11)	−0.0040 (9)	0.0002 (8)	−0.0008 (9)
O5A	0.0301 (10)	0.0236 (9)	0.0199 (9)	0.0087 (8)	−0.0009 (8)	−0.0050 (8)
C5A	0.0217 (11)	0.0157 (11)	0.0147 (11)	−0.0024 (9)	−0.0006 (8)	−0.0033 (9)
O6A	0.0255 (9)	0.0161 (8)	0.0145 (8)	−0.0054 (7)	0.0011 (6)	−0.0018 (6)
C6A	0.0246 (12)	0.0197 (11)	0.0197 (12)	−0.0078 (9)	0.0005 (9)	−0.0054 (9)
O7A	0.0259 (9)	0.0160 (8)	0.0188 (8)	0.0021 (7)	−0.0040 (7)	−0.0038 (7)
C7A	0.0158 (10)	0.0153 (10)	0.0109 (10)	−0.0015 (8)	−0.0014 (8)	−0.0007 (8)
O8A	0.0221 (9)	0.0177 (8)	0.0212 (9)	0.0011 (7)	−0.0076 (7)	0.0001 (7)
C8A	0.0149 (10)	0.0152 (10)	0.0131 (10)	−0.0006 (8)	0.0029 (8)	−0.0001 (8)
C9A	0.0178 (11)	0.0208 (11)	0.0169 (11)	0.0004 (9)	0.0030 (9)	0.0005 (9)
C10A	0.0210 (11)	0.0152 (10)	0.0171 (11)	−0.0028 (9)	−0.0028 (9)	−0.0033 (9)
N1B	0.0150 (9)	0.0152 (9)	0.0138 (9)	−0.0004 (7)	−0.0006 (7)	−0.0013 (8)
C1B	0.0148 (10)	0.0182 (11)	0.0155 (11)	−0.0021 (8)	−0.0011 (8)	0.0003 (8)
O2B	0.0199 (8)	0.0157 (8)	0.0160 (8)	0.0030 (6)	−0.0002 (6)	−0.0006 (7)
C2B	0.0144 (10)	0.0159 (11)	0.0153 (11)	−0.0014 (8)	−0.0012 (8)	−0.0003 (9)
O3B	0.0170 (8)	0.0211 (8)	0.0171 (8)	−0.0072 (7)	−0.0007 (7)	−0.0023 (7)
C3B	0.0129 (10)	0.0155 (10)	0.0158 (11)	−0.0023 (8)	−0.0003 (8)	−0.0005 (8)
O4B	0.0236 (9)	0.0201 (9)	0.0236 (9)	−0.0073 (7)	−0.0063 (7)	0.0068 (7)
C4B	0.0138 (10)	0.0173 (11)	0.0156 (11)	−0.0016 (8)	−0.0004 (8)	0.0008 (8)
O5B	0.0153 (8)	0.0230 (9)	0.0242 (9)	−0.0002 (7)	−0.0014 (7)	0.0055 (7)
C5B	0.0162 (11)	0.0234 (12)	0.0142 (11)	−0.0032 (9)	−0.0023 (8)	−0.0011 (9)
O6B	0.0180 (8)	0.0213 (8)	0.0174 (8)	−0.0063 (6)	−0.0033 (6)	0.0001 (7)
C6B	0.0202 (11)	0.0194 (11)	0.0200 (12)	−0.0037 (9)	−0.0045 (9)	−0.0029 (9)
O7B	0.0320 (10)	0.0188 (8)	0.0197 (9)	0.0081 (7)	0.0022 (7)	0.0017 (7)
C7B	0.0181 (11)	0.0168 (11)	0.0116 (10)	0.0008 (8)	−0.0008 (8)	−0.0003 (8)
O8B	0.0396 (12)	0.0237 (9)	0.0224 (10)	0.0062 (8)	0.0097 (8)	−0.0014 (7)
C8B	0.0178 (11)	0.0159 (10)	0.0176 (11)	0.0023 (9)	−0.0018 (9)	−0.0028 (9)
C9B	0.0194 (12)	0.0299 (13)	0.0225 (12)	0.0021 (10)	−0.0069 (9)	−0.0063 (10)
C10B	0.0266 (13)	0.0206 (12)	0.0173 (11)	0.0007 (10)	0.0018 (9)	0.0028 (9)

### Geometric parameters (Å, º)

**Table:**

O2A—C2A	1.399 (3)	O3B—H3OB	0.86 (4)
O3A—C3A	1.431 (3)	C4A—H4A	1.0000
O4A—C4A	1.435 (3)	O4B—H4OB	0.84 (6)
O6A—C2A	1.415 (3)	C5A—H5A	1.0000
O6A—C6A	1.440 (3)	O5B—H5OB	0.83 (4)
O7A—C8A	1.250 (3)	C6A—H6A2	0.9900
O8A—C8A	1.259 (3)	C6A—H6A1	0.9900
N1A—C1A	1.492 (3)	C9A—H9A3	0.9800
N1A—C7A	1.510 (3)	C9A—H9A1	0.9800
O2A—H2OA	0.82 (4)	C9A—H9A2	0.9800
O3A—H3OA	0.78 (4)	C10A—H10C	0.9800
O4A—H4OA	0.83 (4)	C10A—H10B	0.9800
O5A—H5OA	0.76 (4)	C10A—H10A	0.9800
C1A—C2A	1.526 (3)	C1B—C2B	1.535 (3)
N1A—H1NA	0.87 (3)	N1B—H2NB	0.91 (3)
N1A—H2NA	0.98 (3)	N1B—H1NB	0.90 (4)
C2A—C3A	1.536 (3)	C2B—C3B	1.541 (3)
O2B—C2B	1.398 (3)	C3B—C4B	1.517 (3)
C3A—C4A	1.524 (3)	C4B—C5B	1.540 (3)
O3B—C3B	1.423 (3)	C5B—C6B	1.512 (4)
C4A—C5A	1.535 (3)	C7B—C10B	1.526 (3)
O4B—C4B	1.427 (3)	C7B—C8B	1.551 (3)
C5A—C6A	1.519 (3)	C7B—C9B	1.535 (3)
O5B—C5B	1.431 (3)	C1B—H1B1	0.9900
O6B—C2B	1.418 (3)	C1B—H1B2	0.9900
O6B—C6B	1.437 (3)	C3B—H3B	1.0000
C7A—C8A	1.541 (3)	C4B—H4B	1.0000
C7A—C10A	1.529 (3)	C5B—H5B	1.0000
C7A—C9A	1.531 (3)	C6B—H6B1	0.9900
O7B—C8B	1.262 (3)	C6B—H6B2	0.9900
O8B—C8B	1.240 (3)	C9B—H9B1	0.9800
C1A—H1A1	0.9900	C9B—H9B2	0.9800
C1A—H1A2	0.9900	C9B—H9B3	0.9800
N1B—C1B	1.500 (3)	C10B—H10D	0.9800
N1B—C7B	1.517 (3)	C10B—H10E	0.9800
O2B—H2OB	0.83 (3)	C10B—H10F	0.9800
C3A—H3A	1.0000		
			
C2A—O6A—C6A	112.33 (18)	C7A—C10A—H10C	109.00
C1A—N1A—C7A	115.41 (17)	C7A—C10A—H10A	110.00
C2A—O2A—H2OA	112 (2)	C7A—C10A—H10B	109.00
C3A—O3A—H3OA	104 (3)	H10A—C10A—H10B	109.00
C4A—O4A—H4OA	107 (3)	H10A—C10A—H10C	109.00
C5A—O5A—H5OA	107 (3)	H10B—C10A—H10C	109.00
C7A—N1A—H1NA	106 (2)	C7B—N1B—H2NB	107 (2)
C7A—N1A—H2NA	107.4 (19)	C7B—N1B—H1NB	111 (2)
H1NA—N1A—H2NA	115 (3)	H2NB—N1B—H1NB	112 (3)
N1A—C1A—C2A	108.99 (18)	N1B—C1B—C2B	111.65 (18)
C1A—N1A—H1NA	108 (2)	C1B—N1B—H2NB	106.7 (19)
C1A—N1A—H2NA	105.3 (18)	C1B—N1B—H1NB	106 (2)
O2A—C2A—O6A	107.46 (18)	O2B—C2B—O6B	113.03 (17)
O6A—C2A—C1A	105.52 (16)	O6B—C2B—C1B	102.24 (17)
O6A—C2A—C3A	109.62 (16)	O6B—C2B—C3B	108.88 (17)
C1A—C2A—C3A	109.73 (18)	C1B—C2B—C3B	112.86 (16)
O2A—C2A—C1A	110.90 (17)	O2B—C2B—C1B	112.22 (18)
O2A—C2A—C3A	113.29 (17)	O2B—C2B—C3B	107.61 (18)
O3A—C3A—C2A	107.71 (16)	O3B—C3B—C2B	111.53 (18)
O3A—C3A—C4A	111.05 (17)	O3B—C3B—C4B	112.03 (18)
C2A—C3A—C4A	109.56 (18)	C2B—C3B—C4B	110.23 (16)
O4A—C4A—C3A	107.02 (18)	O4B—C4B—C3B	111.32 (17)
C3A—C4A—C5A	110.21 (17)	C3B—C4B—C5B	110.08 (18)
O4A—C4A—C5A	112.35 (17)	O4B—C4B—C5B	108.48 (19)
O5A—C5A—C4A	112.39 (19)	O5B—C5B—C4B	110.2 (2)
O5A—C5A—C6A	108.72 (18)	O5B—C5B—C6B	108.56 (19)
C4A—C5A—C6A	109.36 (18)	C4B—C5B—C6B	109.76 (19)
O6A—C6A—C5A	111.20 (19)	O6B—C6B—C5B	113.0 (2)
C2B—O6B—C6B	113.45 (17)	N1B—C7B—C8B	108.36 (17)
N1A—C7A—C8A	108.17 (16)	C8B—C7B—C9B	107.71 (18)
C8A—C7A—C10A	113.58 (19)	C8B—C7B—C10B	113.61 (19)
C9A—C7A—C10A	111.62 (18)	C9B—C7B—C10B	110.7 (2)
N1A—C7A—C9A	107.01 (18)	N1B—C7B—C9B	106.01 (19)
N1A—C7A—C10A	108.96 (16)	N1B—C7B—C10B	110.12 (17)
C8A—C7A—C9A	107.24 (18)	O8B—C8B—C7B	116.38 (19)
O7A—C8A—O8A	126.3 (2)	O7B—C8B—O8B	127.0 (2)
O7A—C8A—C7A	117.51 (19)	O7B—C8B—C7B	116.61 (19)
O8A—C8A—C7A	116.13 (18)	N1B—C1B—H1B1	109.00
N1A—C1A—H1A2	110.00	N1B—C1B—H1B2	109.00
C2A—C1A—H1A1	110.00	C2B—C1B—H1B1	109.00
C2A—C1A—H1A2	110.00	C2B—C1B—H1B2	109.00
H1A1—C1A—H1A2	108.00	H1B1—C1B—H1B2	108.00
N1A—C1A—H1A1	110.00	O3B—C3B—H3B	108.00
C1B—N1B—C7B	115.17 (17)	C2B—C3B—H3B	108.00
C2B—O2B—H2OB	110 (2)	C4B—C3B—H3B	108.00
O3A—C3A—H3A	109.00	O4B—C4B—H4B	109.00
C4A—C3A—H3A	109.00	C3B—C4B—H4B	109.00
C2A—C3A—H3A	110.00	C5B—C4B—H4B	109.00
C3B—O3B—H3OB	107 (3)	O5B—C5B—H5B	109.00
O4A—C4A—H4A	109.00	C4B—C5B—H5B	109.00
C5A—C4A—H4A	109.00	C6B—C5B—H5B	109.00
C3A—C4A—H4A	109.00	O6B—C6B—H6B1	109.00
C4B—O4B—H4OB	113 (3)	O6B—C6B—H6B2	109.00
C4A—C5A—H5A	109.00	C5B—C6B—H6B1	109.00
O5A—C5A—H5A	109.00	C5B—C6B—H6B2	109.00
C6A—C5A—H5A	109.00	H6B1—C6B—H6B2	108.00
C5B—O5B—H5OB	110 (3)	C7B—C9B—H9B1	109.00
C5A—C6A—H6A2	109.00	C7B—C9B—H9B2	110.00
O6A—C6A—H6A2	109.00	C7B—C9B—H9B3	109.00
O6A—C6A—H6A1	109.00	H9B1—C9B—H9B2	109.00
C5A—C6A—H6A1	109.00	H9B1—C9B—H9B3	109.00
H6A1—C6A—H6A2	108.00	H9B2—C9B—H9B3	110.00
C7A—C9A—H9A2	109.00	C7B—C10B—H10D	109.00
C7A—C9A—H9A1	109.00	C7B—C10B—H10E	110.00
H9A1—C9A—H9A3	109.00	C7B—C10B—H10F	109.00
C7A—C9A—H9A3	109.00	H10D—C10B—H10E	109.00
H9A1—C9A—H9A2	109.00	H10D—C10B—H10F	109.00
H9A2—C9A—H9A3	109.00	H10E—C10B—H10F	109.00
			
C6A—O6A—C2A—O2A	−61.2 (2)	C9A—C7A—C8A—O8A	89.4 (2)
C6A—O6A—C2A—C1A	−179.57 (18)	C10A—C7A—C8A—O7A	147.9 (2)
C6A—O6A—C2A—C3A	62.3 (2)	N1A—C7A—C8A—O7A	26.8 (3)
C2A—O6A—C6A—C5A	−61.7 (2)	N1A—C7A—C8A—O8A	−155.48 (19)
C1A—N1A—C7A—C10A	−53.2 (2)	C1B—N1B—C7B—C10B	56.0 (2)
C1A—N1A—C7A—C8A	70.7 (2)	C1B—N1B—C7B—C8B	−68.8 (2)
C1A—N1A—C7A—C9A	−174.04 (18)	C1B—N1B—C7B—C9B	175.78 (18)
C7A—N1A—C1A—C2A	−163.70 (16)	C7B—N1B—C1B—C2B	163.55 (16)
N1A—C1A—C2A—O6A	64.6 (2)	N1B—C1B—C2B—O6B	178.40 (16)
N1A—C1A—C2A—C3A	−177.35 (16)	N1B—C1B—C2B—C3B	−64.8 (2)
N1A—C1A—C2A—O2A	−51.4 (2)	N1B—C1B—C2B—O2B	57.0 (2)
O2A—C2A—C3A—O3A	−59.4 (2)	O2B—C2B—C3B—O3B	−61.3 (2)
O6A—C2A—C3A—O3A	−179.43 (17)	O6B—C2B—C3B—O3B	175.87 (17)
O6A—C2A—C3A—C4A	−58.5 (2)	O6B—C2B—C3B—C4B	−59.0 (2)
C1A—C2A—C3A—O3A	65.1 (2)	C1B—C2B—C3B—O3B	63.1 (2)
C1A—C2A—C3A—C4A	−173.98 (16)	C1B—C2B—C3B—C4B	−171.81 (19)
O2A—C2A—C3A—C4A	61.5 (2)	O2B—C2B—C3B—C4B	63.8 (2)
O3A—C3A—C4A—C5A	173.44 (18)	O3B—C3B—C4B—C5B	−179.66 (18)
C2A—C3A—C4A—O4A	177.02 (16)	C2B—C3B—C4B—O4B	175.84 (18)
O3A—C3A—C4A—O4A	−64.1 (2)	O3B—C3B—C4B—O4B	−59.3 (2)
C2A—C3A—C4A—C5A	54.6 (2)	C2B—C3B—C4B—C5B	55.5 (2)
O4A—C4A—C5A—C6A	−172.33 (18)	O4B—C4B—C5B—C6B	−173.64 (18)
C3A—C4A—C5A—O5A	67.8 (2)	C3B—C4B—C5B—O5B	67.9 (2)
O4A—C4A—C5A—O5A	−51.5 (2)	O4B—C4B—C5B—O5B	−54.1 (2)
C3A—C4A—C5A—C6A	−53.1 (2)	C3B—C4B—C5B—C6B	−51.6 (2)
O5A—C5A—C6A—O6A	−67.5 (2)	O5B—C5B—C6B—O6B	−68.2 (2)
C4A—C5A—C6A—O6A	55.5 (3)	C4B—C5B—C6B—O6B	52.4 (2)
C6B—O6B—C2B—C3B	60.3 (2)	N1B—C7B—C8B—O7B	−27.2 (3)
C2B—O6B—C6B—C5B	−58.7 (2)	N1B—C7B—C8B—O8B	155.3 (2)
C6B—O6B—C2B—O2B	−59.3 (2)	C9B—C7B—C8B—O7B	87.1 (2)
C6B—O6B—C2B—C1B	179.89 (18)	C9B—C7B—C8B—O8B	−90.4 (3)
C9A—C7A—C8A—O7A	−88.3 (2)	C10B—C7B—C8B—O7B	−149.9 (2)
C10A—C7A—C8A—O8A	−34.4 (3)	C10B—C7B—C8B—O8B	32.6 (3)

### Hydrogen-bond geometry (Å, º)

**Table:**

*D*—H···*A*	*D*—H	H···*A*	*D*···*A*	*D*—H···*A*
N1*A*—H1*NA*···O6*A*	0.86 (3)	2.40 (3)	2.813 (3)	110 (2)
N1*A*—H1*NA*···O7*A*	0.86 (3)	2.30 (3)	2.674 (2)	107 (2)
O2*B*—H2*OB*···O8*A*^i^	0.84 (3)	1.78 (3)	2.596 (3)	165 (3)
N1*A*—H2*NA*···O7*B*	0.98 (3)	1.78 (3)	2.743 (3)	169 (3)
O5*A*—H5*OA*···O2*B*^ii^	0.76 (4)	2.14 (4)	2.886 (3)	168 (4)
O5*B*—H5*OB*···O3*A*^iii^	0.83 (4)	1.99 (4)	2.804 (3)	165 (3)
O2*A*—H2*OA*···O3*A*	0.82 (4)	2.62 (3)	2.847 (2)	97 (3)
O3*A*—H3*OA*···O4*B*^iv^	0.78 (4)	2.08 (4)	2.785 (3)	149 (3)
O4*A*—H4*OA*···O8*A*^v^	0.84 (4)	2.00 (4)	2.822 (3)	170 (4)
O2*A*—H2*OA*···O8*B*	0.82 (4)	1.87 (4)	2.657 (3)	161 (4)
O4*B*—H4*OB*···O3*B*	0.84 (4)	2.51 (4)	2.886 (2)	108 (3)
O4*B*—H4*OB*···O4*A*^vi^	0.84 (5)	2.14 (5)	2.864 (3)	145 (5)
N1*B*—H2*NB*···O7*A*^i^	0.90 (3)	1.91 (3)	2.795 (3)	168 (3)
N1*B*—H1*NB*···O3*B*	0.90 (4)	2.02 (4)	2.800 (3)	144 (3)
N1*B*—H1*NB*···O7*B*	0.90 (4)	2.40 (3)	2.681 (3)	100 (2)
O3*B*—H3*OB*···O5*B*^vii^	0.86 (4)	1.92 (4)	2.717 (3)	154 (4)

Symmetry codes: (i) *x*+1, *y*−1, *z*; (ii) *x*−1, *y*+1, *z*+1; (iii) *x*, *y*, *z*−1; (iv) *x*−1, *y*, *z*+1; (v) *x*, *y*, *z*+1; (vi) *x*+1, *y*, *z*−1; (vii) *x*+1, *y*, *z*.

### Table S1. C13-NMR spectrum and anomeric distribution of *D*-fructose-2-aminoisobutyric acid in D2O

**Table:**

carbon	α-pyranose	β-pyranose	α-furanose	β-furanose	
C1	51.55	51.72	49.81	51.35	
C2	99.08	98.33	104.65	101.85	
C3	73.12	72.21	85.26	80.64	
C4	74.85	72.39	78.69	77.17	
C5	68.74	71.79	85.32	83.78	
C6	65.80	66.68	63.63	64.76	
C7	n.r.	67.00	66.76	66.85	
C8	n.r.	179.35	179.37	179.46	
C9 or C10	24.55	24.66	24.64	24.64	
C9 or C10	24.16	24.47	24.36	24.43	
					References
% for FruAib	*3.0*	*75.6*	*10.1*	*10.4*	This work
% for *D*-Fru	*2.1*	*68.6*	*5.7*	*23.0*	Kaufmann *et al.*, 2016
% for FruGly	*5*	*66*	*15*	*14*	Mossine *et al.*, 1994
% for FruAla	*5.1*	*71.5*	*10.8*	*11.6*	Kaufmann *et al.*, 2016
% for FruPro	*4.2*	*64.8*	*12.9*	*16.9*	Kaufmann *et al.*, 2016
